# Novel zoonotic *Enterocytozoon* and *Encephalitozoon* genotypes in domestic pigeons (*Columba livia domestica*) in Iran: Public health implications

**DOI:** 10.1016/j.crpvbd.2024.100232

**Published:** 2024-11-23

**Authors:** Alireza Sazmand, Monireh Khordadmehr, Zuhal Önder, Ahmad Oryan, Farinaz Jigari-Asl, Farzad Katiraee, Mehdi Namavari, Zahra Bahiraei, Andrew Hemphill, Domenico Otranto

**Affiliations:** a Department of Pathobiology, Faculty of Veterinary Medicine, Bu-Ali Sina University, 651765-8978, Hamedan, Iran; b Department of Pathobiology, Faculty of Veterinary Medicine, University of Tabriz, 516665-1647, Tabriz, Iran; c Department of Parasitology, Faculty of Veterinary Medicine, Erciyes University, Kayseri, 38039, Turkiye; d Department of Pathobiology, School of Veterinary Medicine, Shiraz University, Shiraz, Iran; e Razi Vaccine and Serum Research Institute, Shiraz Branch, Agricultural Research, Education and Extension Organization (AREEO), Tehran, Iran; f Institute of Parasitology, Vetsuisse Faculty, University of Bern, Länggass-Strasse 122, 3012, Bern, Switzerland; g Department of Veterinary Medicine, University of Bari, Valenzano, Bari, 70010, Italy; h Department of Veterinary Clinical Sciences, City University of Hong Kong, SER, China

**Keywords:** Microsporidiosis, Molecular assay, Histopathology, Public health, Iran

## Abstract

To determine the occurrence of microsporidiosis in domestic pigeons in Iran, privately-owned pigeons presenting weight loss and diarrhea were tested through molecular and histopathological methods. Multiplex-PCR findings showed 57%, 30%, and 14% positivity for *Enterocytozoon*, *Encephalitozoon*, and mixed infection in the fecal samples, respectively. A novel *Enterocytozoon bieneusi* genotype, named IrnEb1, falling into zoonotic group 1 of *E. bieneusi*, and a novel genotype of *Encephalitozoon hellem*, named Irn2E, clustering as a sister taxon to genotype 2B were identified in pigeons for the first time using Sanger sequencing and phylogenetic analyses. Histopathological examination revealed the occurrence of non-suppurative enteritis, nephritis, pneumonia, hepatitis, and encephalitis associated with focal necrosis and inflammatory cell infiltration. Data shown herein present a high prevalence of microsporidiosis in pigeons in Iran. Considering that both detected microsporidian species are zoonotic parasites, these findings suggest that the infected pigeons could pose a risk to public health.

## Highlights

• Multiplex PCR assay targeting the SSU rRNA gene of *Enterocytozoon* and *Encephalitozoon* in pigeon fecal samples.
• DNA of *Enterocytozoon* and *Encephalitozoon*, detected in 57% and 30% of pigeons, respectively.
• A novel genotype of *Encephalitozoon hellem*, named Irn2E, clustering as a sister taxon to genotype 2B was identified.
• A novel genotype of *Enterocytozoon bieneusi*, named IrnEb1, falling into zoonotic group 1 of *E. bieneusi* was identified.
• The high prevalence of microsporidiosis suggests that infected pigeons could pose a risk to public health.

## Graphical abstract

**Figure:**
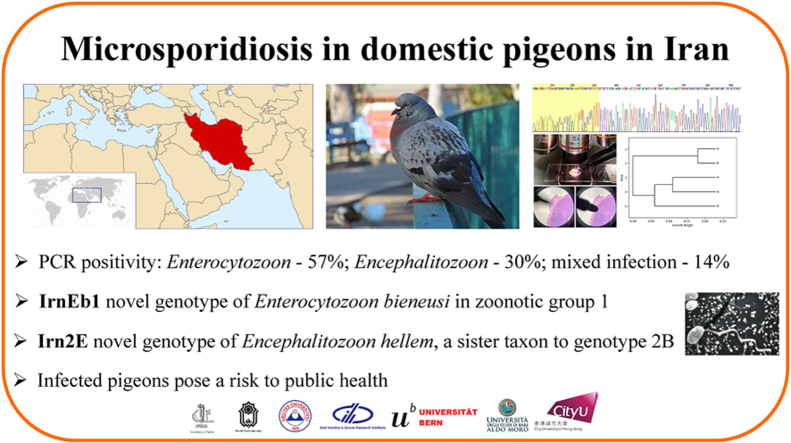

## 1 Introduction

Microsporidia are spore-forming organisms consisting of more than 220 genera with approximately 1700 species. These fungal parasites infect humans, animals, and microeukaryotes (Han et al., 2021) and may be transmitted by food and water, posing a significant threat to humans, wildlife, and economically important species (Li et al., 2019). For example, microsporidiosis is becoming increasingly recognized as an opportunistic infection in immunodeficient patients, such as AIDS patients, organ-transplant recipients, children, travelers, contact lens wearers, and the elderly (Didier, 2005). Ten genera and 17 species of microsporidia have been reported as agents causing human infections (Li et al., 2019). Several of these species, especially species of *Encephalitozoon* and *Enterocytozoon*, are also found in a large variety of birds, being of zoonotic concern (Lallo et al., 2012). Despite the importance of birds as a source of propagation of both microsporidia in the environment, either acting as mechanical vectors or developing active infection (Kicia et al., 2022), this group of animals has received less attention in comparison with mammals.

*Enterocytozoon bieneusi* is the most common cause of human microsporidiosis (Matos et al., 2012), which usually presents with chronic diarrhea, wasting syndrome, cholangitis, rhinitis, and bronchitis (Han et al., 2021; Bojko et al., 2022). Although prevalent in human populations, *E*. *bieneusi* also infects a wide range of birds worldwide, with prevalence ranging from 1.7% to 28.9% (Haro et al., 2005; Lobo et al., 2006; Lallo et al., 2012; Słodkowicz-Kowalska et al., 2013; Pirestani et al., 2013; Tavalla et al., 2018). However, the role of animals in the transmission of this parasite to humans and its importance in public health remains poorly understood (Li et al., 2019).

Over 600 *E. bieneusi* genotypes have been identified in humans and animals based on sequence analysis of the internal transcribed spacer (ITS) region of the rRNA gene (Zhang et al., 2021). Phylogenetic comparative analysis has determined that these genotypes cluster into 15 distinct genetic groups with different host ranges, with human-pathogenic genotypes included in groups 1 and 2, and host-adapted genotypes included in the remaining groups 3–15 (Li et al., 2020; Jiang et al., 2024). Group 1 genotypes including A, D, EbpA, Peru6, Peru 8, Peru6-var, Peru6-var, BEB6, Henan-IV, Peru11, and Type IV and group 2, 5, and 10 genotypes of *E. bieneusi* are reported in free-ranging, captive, and domestic birds (Deng et al., 2019; Feng et al., 2019; Li et al., 2019; Pekmezci et al., 2020, 2021; Wang et al., 2020). The D, M, J, Col01-Col07, Peru6, Peru6-var, and EpbA genotypes of *E. bieneusi* are identified in pigeons worldwide (Haro et al., 2005; Lobo et al., 2006; Lallo et al., 2012; Pirestani et al., 2013; Zhao et al., 2016; Pekmezci et al., 2021). Type IV genotype of *E. bieneusi* has a wide range of hosts, including non-human primates, domesticated animals, and avian species, and is regarded as having zoonotic potential (Deng et al., 2019; Ruan et al., 2021).

*Encephalitozoon hellem*, on the other hand, is the most prevalent *Encephalitozoon* species in free-range, captive, and livestock birds, including those in the orders Psittaciformes, Passeriformes, Anseriformes, Gruiformes, Columbiformes, and Struthioniformes, which may act as zoonotic reservoirs (Kasicková et al., 2009; Hinney et al., 2016; Itoh et al., 2021; Seatamanoch et al., 2022). *Encephalitozoon hellem* causes keratoconjunctivitis, sinusitis, bronchitis, nephritis, cystitis, prostatitis, urethritis, pneumonia, and disseminated infection in human patients (Han et al., 2021; Bojko et al., 2022). The majority of the hosts of *E. hellem* are bird species, and studies performed on urban pigeons revealed that there is no barrier to the transmission of this microsporidian between the infected pigeons and humans (Haro et al., 2005). To date, seven *E. hellem* genotypes (1A, 1B, 1C, 2A, 2B, 2C (or 3), and 2D) were identified based on sequence analyses of the ITS, PTP (polar tube protein), small subunit ribosomal RNA gene (SSU rDNA), and IGS (intergenic spacers of ribosomal genes) (Mathis et al., 1999; Xiao et al., 2001; Haro et al., 2003; Galván et al., 2013).

Domestic pigeons (*Columba livia domestica*) are bred worldwide mainly for meat, eggs, and leisure (Harlin, 1994). Pigeons are very popular in Iran; they are kept free or in cages in the backyard or roof of houses and are used for flying competitions, breeding, entertainment, and meat and egg production. They have close contact with humans and other animals, rendering them a potential carrier and reservoir of zoonotic infections (Adang et al., 2008). In a previous study in the Czech Republic, the prevalence of microsporidian infection was higher in pigeons (31.1%) than in other birds (18.8%) (Lallo et al., 2012). In another study, human-associated microsporidia were diagnosed in pigeons from urban parks in Spain (Haro et al., 2005), indicating the importance of pigeons in the epidemiology of microsporidiosis. However, limited information about avian microsporidiosis is available in general, as well as in Iran. Microsporidial infections have been detected by PCR in fecal specimens of pigeons from Tehran and Ahvaz only (Pirestani et al., 2013; Tavalla et al., 2018).

In the present study, we assessed the prevalence and pathology of *Enterocytozoon* and *Encephalitozoon* infections in domestic pigeons from a north-western province bordering Armenia and Azerbaijan by performing histopathological and molecular analyses.

## 2 Materials and methods

### 2.1 Study area

This study was performed in Tabriz city, the capital of East-Azerbaijan Province in north-west Iran (38.0792°N, 46.2887°E, 1351 m above sea level) with a population of 1,643,960 in 2022. It is located in the middle of the Azerbaijan border and the northeastern part of Lake Urmia. The city has a tropical and subtropical steppe climate (Köppen-Geiger classification: BSk) with an average yearly rainfall of *c*.360 mm.

### 2.2 Sample collection

From April to June 2021, a total of 100 domestic pigeons (rock pigeon, *Columba livia domestica*), 50 males and 50 females, 56 over and 44 under six months old, were purchased from different locations in the city. Because there was no information on the prevalence of *Enterocytozoon* and *Encephalitozoon* infections in this region, sampling was done based on a non-probability sampling method (i.e. convenience sampling). Their owners had reported clinical symptoms such as weight loss and diarrhea. Two veterinarians, a pathologist, and an avian diseases specialist, conducted clinical evaluations, and the observed symptoms were recorded in individual data forms. After decapitation, a systematic necropsy was performed, and different organs and tissues were examined for gross lesions (e.g. macroscopic necrosis, vascular hyperemia, congestion, and hemorrhage). Besides, the age and sex of the birds were recorded based on the owner’s information, which was later confirmed at necropsy by observing the development of sexual organs and their size.

Individual fecal samples were collected in Eppendorf microtubes and stored at −20 °C for molecular study. Intestine, brain, liver, kidney, and lung tissues were fixed in a 10% formalin buffer solution for at least 48 h for further histopathology examination. Two longitudinal sections of the intestine and one transverse section of the brain, lungs, liver, and kidneys were then processed routinely, embedded in paraffin, sectioned, and stained using hematoxylin-eosin (H&E). The tissue sections (*n* = 600) were examined under a light microscope Olympus CH-30 (Tokyo, Japan), and the presence of the parasites and probable histopathological lesions, including hyperemia, epithelial alteration, including cell degeneration and necrosis, and inflammatory cell infiltration, were recorded.

### 2.3 DNA extraction and PCR assay

Fecal specimens (*c*.200 mg) were thoroughly homogenized using disposable plastic applicators. Three freeze/thaw cycles were then performed, and finally, the obtained supernatant was used for DNA extraction after centrifugation (12,000×*g* at 4 °C for 20 min). Total genomic DNA (gDNA) was extracted using a commercial DNA extraction kit (MBST, Tehran, Iran) based on the manufacturer’s instructions. This kit has proved to be able to disrupt different microsporidian spores, including *Encephalitozoon* and *Enterocytozoon* (Meftahi et al., 2023; Chamanara et al., 2024), although it would benefit from further validation. Quantitative and qualitative assessments were performed using NanoDrop 2000™ (Thermo Scientific, Waltham, MA, USA) and 0.8% agarose gels.

DNA samples were subjected to a multiplex nested conventional PCR (m-nested PCR) method targeting the small subunit ribosomal RNA (SSU rRNA) gene, which can differentiate *Enterocytozoon* and *Encephalitozoon* based on PCR product size (Katzwinkel-Wladarsch et al., 1996) (Table 1). Amplifications included denaturation (95 °C for 5 min), 35 cycles (denaturation at 94 °C for 45 s; primer annealing at 50 °C for 1 min, and 50 °C for 1 min in first and second nested PCR, respectively; and elongation at 72 °C for 1 min). The final elongation was extended to 10 min. The amplicon size for the *Enterocytozoon* product on the 2% gel electrophoresis was *c.*500 bp (MSP-3/MSP-4B primer pair), and the resulting product for *Encephalitozoon* was *c*.300 bp (MSP-3/MSP-4A primer pair). For all reactions, DNA of *E. cuniculi* (obtained in a previous study by Sadeghi-Dehkordi et al., 2019), *E. bieneusi* (kindly provided by Professor David Modrý, Masaryk University, Czech Republic) as a positive control and distilled deionized water as a negative control were used.

**Table 1:** Primers used in this study to amplify SSU rRNA gene fragments of 500 bp for *Enterocytozoon* and 300 bp for *Encephalitozoon*.

PCR	Primer name	Sequence (5′-3′)
Nest 1	MSP-1	TGAATGKGTCCCTGT
	MSP-2A	TCACTCGCCGCTACT
	MSP-2B	GTTCATTCGCACTACT
Nest 2	MSP-3	GGAATTCACACCGCCCGTCRYTAT
	MSP-4A	CCAAGCTTATGCTTAAGTYMAARGGGT
	MSP-4B	CCAAGCTTATGCTTAAGTCCAGGGAG

To prevent cross-contamination in nested PCR reactions, the preparation of reagents was performed in separate PCR-UV chambers equipped with independent batches of reagents, micropipette sets, and sterile reagent tubes.

### 2.4 Sequencing and phylogenetic analysis

Seven positive-PCR products (3 for *Encephalitozoon* and 4 for *Enterocytozoon*) were randomly selected and sequenced bidirectionally in an Applied Biosystems 3500 Genetic Analyzer (Thermo Fisher Scientific, Waltham, MA, USA). Sequencing of the PCR products was performed with Applied Biosystems 3500 Genetic Analyzer (Thermo Fisher Scientific, Waltham, MA, USA). The raw forward and reverse reads were aligned and assembled using Geneious Prime® 2023.1.2 (https://www.geneious.com) to obtain the consensus sequences. The latter were compared with those available in the GenBank database using the Basic Local Alignment Search Tool (BLAST; http://blast.ncbi.nlm.nih.gov/Blast.cgi).

Phylogenetic analysis of the SSU rRNA gene was performed using the neighbor-joining method (NJ) implemented in MEGA11 (Tamura et al., 2021) to assess the phylogenetic relationships of the microsporidian parasites based on the genetic distance (Kimura-2-parameter model). The bootstrap support values were calculated based on 1000 replicates.

### 2.5 Statistical analyses

To determine the correlations between infections and sex (male/female) or age (under/over six months-old) of the pigeons, the Chi-square test was used. Differences were considered significant at *P* < 0.05. The analyses were performed with IBM SPSS Statistics v.22 software.

## 3 Results

### 3.1 Molecular prevalence and risk factors

DNA of *Enterocytozoon* spp. was detected in 57 pigeons (57%; 95% CI: 47–66%), and DNA of *Encephalitozoon* spp. was observed in 30 pigeons (30%; 95% CI: 21–39%). Simultaneous infection with both genera was observed in 14 birds (14%; 95% CI: 7–20%). There were no significant differences in *Encephalitozoon* infection rate between sexes (female: 17%; male: 13%) and the two age categories (juvenile: 11%; adult: 19%). Similarly, there were no significant differences in *Enterocytozoon* spp. infection rate related to sex (female: 28%; male: 29%). However, a remarkable difference was observed between age categories and *Enterocytozoon* spp. infection rate (juvenile: 33%; adult: 24%) (Table 2).

**Table 2:** Risk factors associated with the infection of pigeons (*n* = 100) with *Enterocytozoon* and *Encephalitozoon*.

Risk factor	Pathogen	No. infected	*χ*^2^	*P-*value
Age	*Enterocytozoon*	33J/24A	9.22	0.002∗∗
	*Encephalitozoon*	11J/19A	0.92	0.33
	Co-infection	7J/7A	0.61	0.43
Sex	*Enterocytozoon*	28F/29M	0.16	0.68
	*Encephalitozoon*	17F/13M	0.18	0.66
	Co-infection	8F/6M	0.10	0.74

*Abbreviations*: A, adult (over six months-old); J, juvenile (under six months-old); F, female; M, male.

*Note*: ∗∗ Significant difference (*P* < 0.01).

### 3.2 Sequence analyses

Consensus sequences for the detected pathogens displayed 83.8–97.74% and 85–100% nucleotide identity and query cover, respectively, with the sequences available in the GenBank database. The PCR-positive products from all *E. hellem* and *E. bieneusi* were successfully sequenced, and the sequences were 267 bp and 485 bp in length, respectively.

BLAST and multiple alignment analyses of the SSU rDNA sequences of our isolates revealed novel genotypes of *E. hellem* (Irn2E; *n* = 3) and *E. bieneusi* (IrnEb1; *n* = 4). The genotype Irn2E of *E. hellem* has four single nucleotide polymorphisms (SNPs) when compared to the genotype 2B (GenBank: AF338368, from a human in the USA; Supplementary Fig. S1). The genotype IrnEb1 of *E. bieneusi* had four nucleotide differences compared with genotype Type IV (GenBank: AF267141, from a cat; 99.17% homology; Supplementary Fig. S2). The sequences were deposited in GenBank under the accession numbers OQ799983-OQ799985 (*E. hellem* Irn2E genotype) and OQ800855-OQ800858 (*E. bieneusi* IrnEb1 genotype).

### 3.3 Phylogenetic relationships of the novel genotypes of *E. hellem* and *E. bieneusi*

**Fig. 1:**
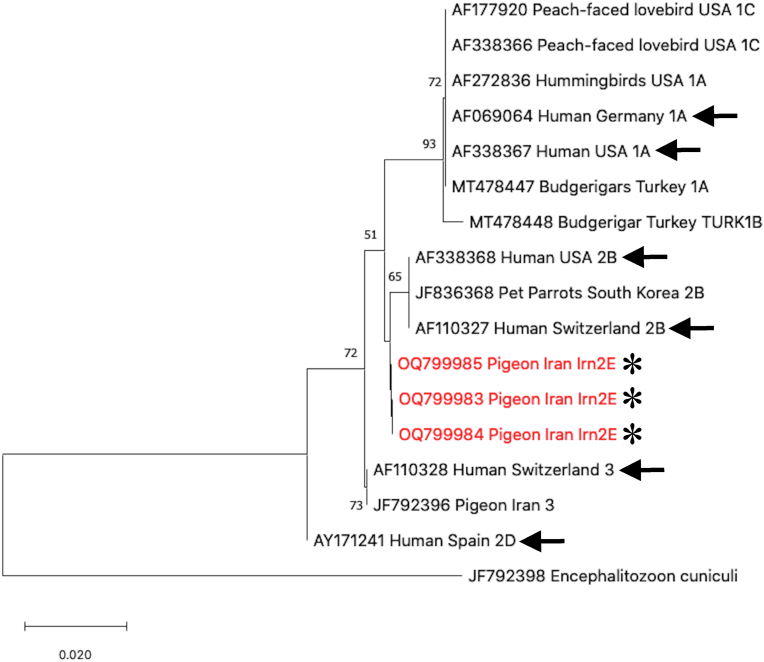
Phylogenetic trees based on NJ analysis of SSU rRNA gene sequences and showing the relationships of the novel *Encephalitozoon hellem* genotype Irn2E identified from pigeons in our study and other genotypes from various hosts in different countries deposited in GenBank. Genotypes identified in this study are indicated with asterisks, and genotypes isolated from humans are indicated with arrows. Numbers at the nodes represent the bootstrap values based on 1000 replicates. *Encephalitozoon cuniculi* genotype (GenBank: JF792398) from a pigeon in Iran was used as the outgroup.

**Fig. 2:**
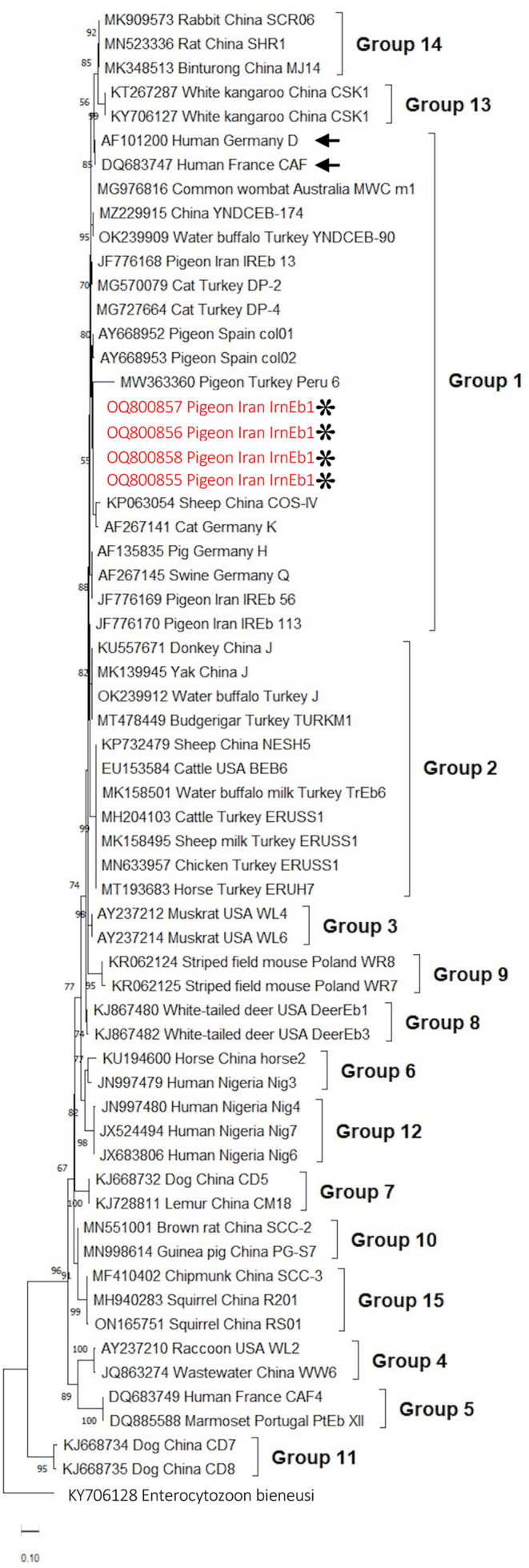
Phylogenetic trees based on NJ analysis of SSU rRNA gene sequences and showing the relationships of the novel *Enterocytozoon bieneusi* genotype IrnEb1 identified from pigeons in our study and other genotypes from various hosts in different countries deposited in GenBank. Genotypes identified in this study are indicated with asterisks, and genotypes isolated from humans are indicated with arrows. Numbers at the nodes represent the bootstrap values based on 1000 replicates. *Enterocytozoon bieneusi* genotype CSK2 (GenBank: KY706128) from white kangaroo in China was used as the outgroup.

The identified *E. hellem* genotype Irn2E was recovered as a sister taxon to genotype 2B in the NJ tree (Fig. 1). All novel genotypes of *E. bieneusi* clustered within the so-called zoonotic group 1 in the NJ tree, close to an isolate reported from a cat in Germany (GenBank: AF267141) and an isolate from sheep in China (GenBank: KP063054) (Fig. 2).

### 3.4 Clinical findings

The most common clinical signs in the examined birds were weight loss (100%), diarrhea (46%), respiratory distress (19%), and torticollis (4%). Enteritis (35%) associated with hyperemia (16%) or hemorrhage (4%) were the most prevalent recorded necropsy lesions. Other observations included purulent bronchopneumonia followed by focal consolidation and necrosis (5%), macroscopic vascular congestion (7%) and focal hemorrhage (3%) in the brain, and hypertrophy of the kidneys (13%) together with focal hemorrhage (5%).

### 3.5 Pathological findings

**Fig. 3:**
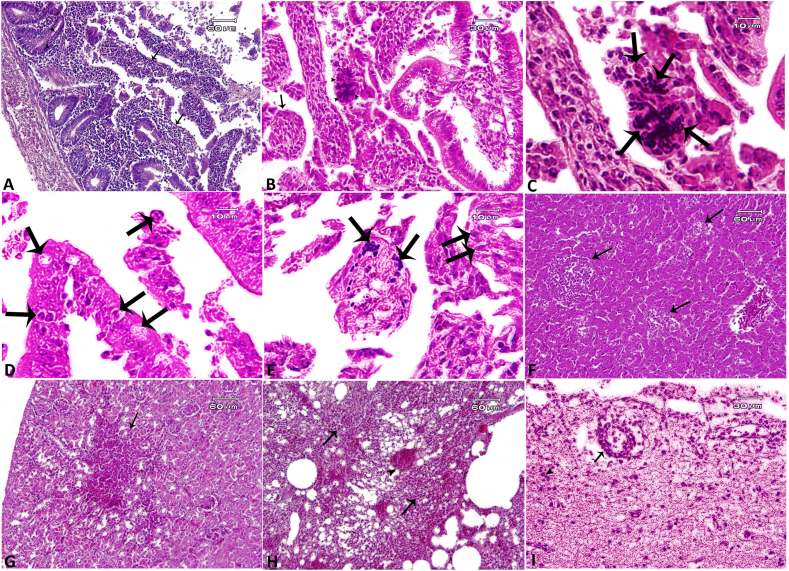
Photomicrographs of pigeons naturally infected with and molecularly positive to microsporidia. **A**, **B** Diffused and mixed inflammatory cell infiltration associated with sloughing of the epithelial cells in the small intestine (*arrows*). Aggregation of microsporidia within the cytoplasm of enterocytes in the intestinal crypt (*arrowheads*). **C** Higher magnification of **B**. **D**, **E** Aggregation of microsporidia within the cytoplasm of enterocytes in the intestinal crypt (*arrows*) in various intracellular stages. **F** Liver: multifocal necrosis and inflammation (*arrows*). **G** Kidney: focal inflammation in the cortex layer (*arrow*). **H** Lung: diffused inflammatory cell infiltration (*arrows*) associated with vascular congestion (*arrowhead*). **I** Brain: section showing perivascular cuffing (PVC) (*arrow*) to gather with focal gliosis. Hematoxylin & eosin staining.

Histopathological examination of the small intestine revealed non-specific lesions such as enteritis consisting of diffused and mixed inflammatory cell infiltration, including lymphocytes, plasma cells, macrophages, and fewer in number heterophils associated with vascular hyperemia and sloughing of the epithelial cells. Enteritis was observed in all of the 30 *Encephalitozoon*-positive (Enc) and in 57 *Enterocytozoon*-positive (Ent) pigeons. Besides, there were focal to multifocal inflammation and necrosis in the tissue sections of the liver (hepatitis: 100% Enc; 93% Ent), kidneys (nephritis: 56.6% Enc; 75.4% Ent), and lungs (pneumonia: 76.6% Enc; 73.7% Ent). Mild focal to diffuse gliosis, perivascular cuffing, and edema in the brain tissues were observed in 6.6% Enc and 15.8% Ent cases (Supplementary Table S1). Aggregation of microsporidia was observed within the cytoplasm of enterocytes in the crypts as well as in the cells of lamina propria of the small intestine, particularly in the ileum sections (Fig. 3).

## 4 Discussion

This study demonstrated that *Enterocytozoon* and *Encephalitozoon* are highly prevalent in pigeons, suggesting that pigeons can influence the epidemiology of these pathogens, posing a potential threat to both humans and animals. However, the high prevalence rates identified in this survey were likely due to the sampled pigeons, all of them presenting weight loss and diarrhea. Hence, lower figures would be expected in apparently healthy animals. This means that pigeon owners handling symptomatic animals could be at higher risk of infection with microsporidian pathogens. Although limited in numbers, both *E. bieneusi* and *E. hellem* have already been reported in pigeons in other regions of Iran (Pirestani et al., 2013; Tavalla et al., 2018; *via* m-nested PCR) and other countries, such as Spain (Haro et al., 2005; *via* SSU rDNA PCR), Portugal (Lobo et al., 2006; *via* ITS PCR), Poland (Słodkowicz-Kowalska et al., 2013; *via* multiplex fluorescent *in situ* hybridization, FISH), Türkiye (Pekmezci et al., 2021; *via* ITS PCR), and Brazil (Lallo et al., 2012; *via* ITS PCR). Pigeons are more susceptible to infections with microsporidia compared to exotic and pet birds (Lallo et al., 2012). Of note, in Spain and the Netherlands, where urban pigeons were diagnosed to be reservoirs of human-associated microsporidia (i.e. *E. bieneusi*, *E. hellem*, and *E. intestinalis*) it was suggested that there is no barrier to microsporidian transmission between park pigeons and humans resulting in a high-risk of microsporidiosis in children and in the elderly visiting urban parks (Haro et al., 2005; Bart et al., 2008). Because bird droppings dry quickly and produce dust, inhalation of dust containing viable spores into the respiratory tract might initiate an infection, especially in immunocompromised persons. A previous study demonstrated that a person with 30 min of occupational or non-occupational exposure to urban feral pigeons, such as exposure through the cleaning of surfaces contaminated with pigeon excrements, could inhale approximately 3.5 × 10^3^ *E. bieneusi* spores and that a nearby person may inhale 1.3 × 10^3^ spores, highlighting the importance of pigeons as a source of air- and water-borne contamination (Graczyk and Cranfield, 2000). Although the minimal infectious dose for microsporidia is unknown, it is comparable to those of other intestinal parasites (i.e. 10–100 spores; see Ford, 1999). Hence, the risk of keeping pigeons in houses should be warned to bird lovers.

The high nucleotide identity of sequences of *E. hellem* Irn2E (i.e. 98.5%) with the reference sequence *E. hellem* 2B (GenBank: AF338368) suggests that this could be a novel genotype. Previous studies in Iran reported genotypes 1A and 3 of *E. hellem* in pigeons of Iran (Pirestani et al., 2013), the Czech Republic (Kasicková et al., 2009), Brazil (Lallo et al., 2012), and Spain (Haro et al., 2005). Although we did not find *E. cuniculi* and *E. intestinalis* in pigeons, both species have been repeatedly reported from pigeons, exotic birds, mice, rats, dogs, rabbits, and immunodeficient human patients in Iran (Delrobaei et al., 2019; Sadeghi-Dehkordi et al., 2019). Furthermore, the novel genotype IrnEb1 was the only *E. bieneusi* genotype in pigeons in the research area, although previous studies reported *E. bieneusi* genotype D as the predominant genotype in both humans and animals in Iran (Mirjalali et al., 2014; Javanmard et al., 2018; Delrobaei et al., 2019). Since the present data rely on a limited number of SSU rDNA sequences, genotyping more samples from pigeons and multilocus phylogeny analysis (Li and Xiao, 2019; Valenčáková and Sučik, 2020; Pekmezci et al., 2021) will increase our knowledge regarding the population structure of microsporidians in pigeons and the public health importance of microsporidiosis.

Although molecular positivity for microsporidia was associated with different pathologies in pigeons of this study, especially in the *E. hellem* infection (Supplementary Table S1), the described clinical symptoms of microsporidiosis in birds (e.g. diarrhea, hypoxia, and respiratory distress) are not considered specific (Vergneau-Grosset and Larrat, 2015). Hence, the observed pathologies cannot be directly associated with microsporidiosis since the microsporidia-infected pigeons could be co-infected with other microbes or protozoan parasites such as *Cryptosporidium* spp., *Toxoplasma gondii*, *Neospora caninum*, and *Sarcocystis* spp. (Khordadmehr et al., 2023; Supplementary Table S2). Furthermore, neurological signs recorded in one out of 30 and two out of 57 PCR-positive pigeons for *E. bieneusi* and *E. hellem*, respectively, were again, not pathognomonic for microsporidiosis. In this regard, in a study comparing the neurological signs of *E. cuniculi*-infected rabbits (i.e. *Oryctolagus cuniculus*) to their postmortem lesions, no correlation was reported between the severity of clinical signs and histopathological lesions (Kunstýr et al., 1986; Csokai et al., 2009). Similarly, it was shown that the intensity of microsporidia fecal shedding, and thus the sensitivity of fecal screening, is correlated with the severity of infection by intestinal microsporidia in humans but is not correlated with the occurrence of diarrhea (Clarridge et al., 1996). Experimental studies are needed to clarify whether this phenomenon also occurs in other hosts, including pigeons.

The main reported histopathological lesions in the microsporidian infection were non-suppurative encephalitis, chronic lymphoplasmacytic granulomatous interstitial nephritis, pneumonia, myocarditis, and hepatitis in kidneys, lungs, hearts, and livers (Percy and Barthold, 2007; Gruber et al., 2009; Künzel and Joachim, 2010). Indeed, microsporidia can infect various types of host cells such as macrophages, histiocytes, epithelial cells (e.g. renal tubules, urinary tract, epithelial cells of the small intestine, conjunctiva and cornea, bile ducts and gallbladder, bronchi, nasal mucosa, and uterus), endothelial cells of blood vessels and numerous other types of cells (Orenstein, 2003); hence, histological lesions can be observed in most tissues of the affected hosts. Similar to our findings in pigeons, randomly distributed foci of necrosis associated with the presence of macrophages and few to numerous heterophils were detected in the liver tissue sections in budgerigars (Black et al., 1997). However, in contrast to the study on budgerigars in which little to no inflammation was associated with the organisms in the intestine (Black et al., 1997), we observed the parasites within the cytoplasm of enterocytes in the intestinal crypt of infected pigeons, which also showed enteritis. Indeed, all of the PCR-positive pigeons in the present study showed enteritis and intestinal lesions. Further quantitative molecular-based studies of different tissues and organs of pigeons are recommended to elucidate the severity of the pathology and the distribution of the parasites in different organ tissues of birds.

This study has some limitations. Thus, only seven PCR-positive products (3 for *Encephalitozoon* and 4 for *Enterocytozoon*) could be Sanger sequenced because of the low budget; more sequenced isolates could have provided a clearer picture of the epidemiological context. Another limitation was that we did not conduct PCR for detection of microsporidia in other tissue samples (e.g. intestine, brain, liver, kidney, lung) to confirm extra-intestinal dissemination in the cases where histopathology did not show clear results. Finally, it should be noted that the tissues of pigeons in this study were previously screened for *Toxoplasma gondii*, *Neospora caninum*, *Sarcocystis* spp., and *Cryptosporidium* spp., and found to be simultaneously infected with the examined parasites (Supplementary Table S2) hence, clinical findings might not be directly linked with microsporidiosis.

## 5 Conclusion

Herein, *Enterocytozoon bieneusi* and *Encephalitozoon hellem* were detected as prevalent zoonotic microsporidian species with a considerable infection rate in pigeons. Since pigeons seem to be more sensitive to microsporidiosis compared to other exotic and pet birds and may act as mechanical vectors or replicators of these pathogens, the environmental and public health significance of pigeons should be further investigated. Broadcasting these findings to the public and public health policymakers is suggested.

## CRediT authorship contribution statement

**Alireza Sazmand:** Conceptualization, Investigation, Writing – original draft, Writing – review & editing, Supervision, Resources. **Monireh Khordadmehr:** Conceptualization, Investigation, Writing – original draft, Writing – review & editing, Supervision, Resources, Project administration, Funding acquisition. **Zuhal Önder:** Investigation, Writing – original draft, Writing – review & editing. **Ahmad Oryan:** Writing – review & editing. **Farinaz Jigari-Asl:** Investigation. **Farzad Katiraee:** Investigation. **Mehdi Namavari:** Investigation. **Zahra Bahiraei:** Investigation. **Andrew Hemphill:** Conceptualization, Writing – review & editing. **Domenico Otranto:** Conceptualization, Writing – review & editing.

## Ethical approval

All applicable international, national, and institutional guidelines for the care and use of animals were followed. Sampling was approved by the Animal Research Ethics Committee of the University of Tabriz (ID: IR.TABRIZU.REC.1400.004).

## Funding

This work was supported by the 10.13039/501100007831University of Tabriz, International and Academic Cooperation Directorate, in the framework of the TabrizU-300 programme. The funder had no role in the study design, analysis, decision to publish, or manuscript preparation.

## Declaration of competing interest

The authors declare that they have no known competing financial interests or personal relationships that could have appeared to influence the work reported in this paper.

## Data Availability

Data supporting the conclusions of this article are included within the article and its supplementary file. The newly generated sequences were deposited in the GenBank database under the accession numbers OQ799983-OQ799985 (*Encephalitozoon hellem Irn2E genotype), and OQ800855-OQ800858 (Enterocytozoon bieneusi IrnEb1 genotype).*
