# Supplementary material for: Novel zoonotic Enterocytozoon and Encephalitozoon genotypes in domestic pigeons (Columba livia domestica) in Iran: Public health implications
Source: Curr Res Parasitol Vector Borne Dis. 2024 Nov 23;7:100232. doi: 10.1016/j.crpvbd.2024.100232 (PMC11638640; doi:10.1016/j.crpvbd.2024.100232)
Supplement: Multimedia component 1 [file mmc1.pdf]

Novel zoonotic *Enterocytozoon* and *Encephalitozoon* genotypes in domestic pigeons (*Columba livia domestica*) in Iran: Public health implications

Alireza Sazmand [a#**], Monireh Khordadmehr [b#*], Zuhal Önder [c], Ahmad Oryan [d], Farinaz Jigari-Asl [b], Farzad Katiraee [b], Mehdi Namavari [e], Zahra Bahiraei [a], Andrew Hemphill [f], Domenico Otranto [g,h]

Supplementary file 1

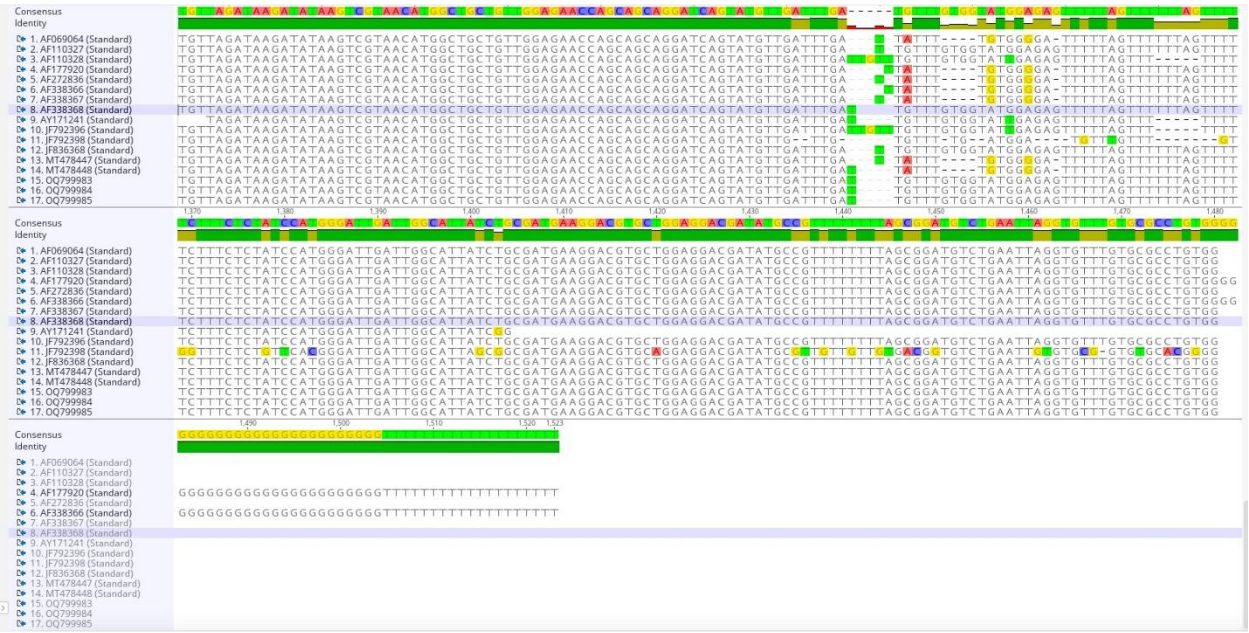

Supplementary Figure S1. *Encephalitozoon hellem* alignment. The genotype of *E. hellem* Irn2E (*n* = 3) has four single nucleotide polymorphisms (SNPs) when compared to the genotype 2B (GenBank: AF338368; from a human in the USA).

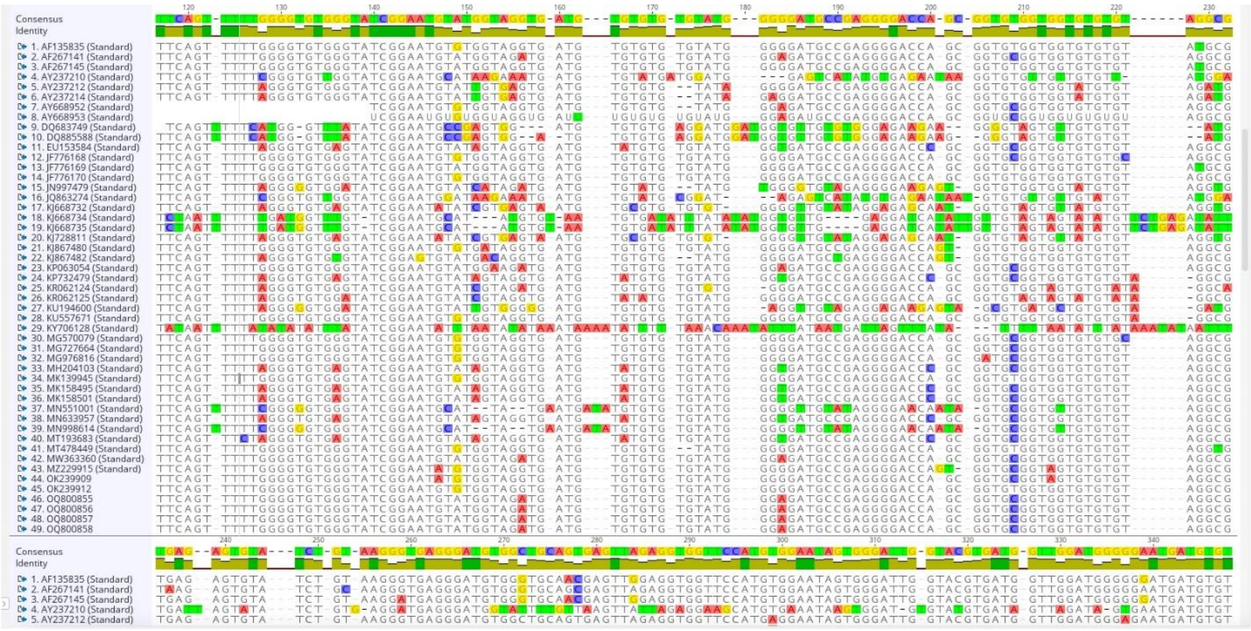

Supplementary Figure S2. *Enterocytozoon bieneusi* alignment. *E. bieneusi* IrnEb1 genotype (n = 4) has four nucleotide differences compared with genotype Type IV from a cat (GenBank: AF267141; 99.17% homology).

Supplementary Table S1. The main pathological findings observed in pigeons PCR-positive for *Encephalitozoon* (*n* = 30) and *Enterocytozoon* (*n* = 57).

**Lesion**	***Encephalitozoon* (*n* = 30)**	χ^2	***P*-value**	***Enterocytozoon* (*n* = 57)**	χ^2	***P*-value**
Diarrhea, *n* (%)	11 (36.7)	0.018	0.89	18 (31.6)	3.95	**0.047***
Enteritis, *n* (%)	30 (100)	16.5	**0.0***	57 (100)	49.00	**0.0***
Hepatitis, *n* (%)	30 (100)	18.18	**0.0***	53 (93)	41.90	**0.0***
Pneumonia, *n* (%)	23 (76.6)	10.05	**0.002***	42 (73.7)	17.92	**0.0***
Nephritis, *n* (%)	17 (56.6)	0.75	0.38	43 (75.4)	36.16	**0.0***
Encephalitis, *n* (%)**	2 (6.6)	1.25	0.263	9 (15.8)	6.76	**0.009***

* Significant difference.
** Perivascular cuffing with inflammatory cell infiltration (1/30 and 2/57) and gliosis (1/30 and 7/57) are considered.

Histopathological examination of the small intestine revealed, enteritis consisting of diffused and mixed inflammatory cell infiltration including lymphocytes, plasma cells, macrophages, and fewer neutrophils associated with vascular hyperemia and sloughing of the epithelial cells. Enteritis was observed in 100% of the 30 *Encephalitozoon*-positive (Enc) and 57 *Enterocytozoon*-positive (Ent) pigeons. Besides, there were focal to multifocal inflammation and necrosis in the tissue sections of the liver (hepatitis; 100% Enc, 93% Ent), kidneys (nephritis; 56.6% Enc, 75.4% Ent), and lungs (pneumonia: 76.6% Enc, 73.7% Ent). Mild focal to diffuse gliosis, perivascular cuffing, and edema in the brain tissues were observed in 6.6% Enc and 15.8% Ent cases.

Supplementary Table S2. Pathogens detected in the tissues of domestic pigeons (*Columba livia domestica*) in Iran (*n* = 100).

Case No.	***Enterocytozoon* (this study)**	***Encephalitozoon* (this study)**	***Toxoplasma* (Reference #1)**	***Neospora* (Reference #1)**	***Sarcocystis* (Reference #1)**	***Cryptosporidium* (Reference #2)**
1	Negative	Negative	Negative	Negative	Negative	Positive
2	Negative	Negative	Negative	Negative	Negative	Positive
3	Negative	Negative	Positive	Negative	Negative	Positive
4	Negative	Negative	Negative	Negative	Negative	Positive
5	Positive	Negative	Positive	Negative	Negative	Positive
6	Negative	Negative	Negative	Negative	Negative	Positive
7	Negative	Negative	Negative	Negative	Negative	Positive
8	Negative	Positive	Negative	Negative	Negative	Positive
9	Negative	Negative	Negative	Negative	Negative	Positive
10	Negative	Negative	Negative	Negative	Negative	Positive
11	Negative	Negative	Negative	Negative	Negative	Positive
12	Negative	Positive	Negative	Negative	Negative	Positive
13	Negative	Positive	Negative	Negative	Negative	Positive
14	Positive	Negative	Negative	Negative	Negative	Positive
15	Positive	Negative	Negative	Negative	Negative	Positive
16	Negative	Negative	Positive	Negative	Negative	Positive
17	Positive	Negative	Negative	Negative	Negative	Positive
18	Positive	Negative	Negative	Negative	Negative	Positive
19	Negative	Positive	Negative	Negative	Negative	Positive
20	Positive	Positive	Negative	Negative	Negative	Positive
21	Negative	Negative	Negative	Negative	Negative	Positive
22	Negative	Negative	Negative	Negative	Negative	Positive
23	Positive	Negative	Negative	Negative	Negative	Positive
24	Negative	Positive	Negative	Negative	Negative	Positive
25	Positive	Negative	Negative	Negative	Negative	Positive
26	Positive	Positive	Positive	Negative	Negative	Positive
27	Positive	Negative	Positive	Negative	Negative	Positive
28	Positive	Positive	Negative	Negative	Negative	Positive
29	Positive	Positive	Negative	Negative	Negative	Positive
30	Positive	Negative	Negative	Negative	Negative	Positive
31	Positive	Negative	Negative	Negative	Negative	Positive
32	Positive	Positive	Negative	Negative	Negative	Positive
33	Positive	Negative	Negative	Negative	Negative	Positive
34	Negative	Negative	Negative	Negative	Negative	Positive
35	Positive	Negative	Negative	Negative	Negative	Positive
36	Negative	Negative	Negative	Negative	Negative	Positive
37	Positive	Negative	Positive	Negative	Negative	Positive
38	Negative	Positive	Positive	Negative	Negative	Positive
39	Positive	Positive	Positive	Negative	Negative	Positive
40	Negative	Negative	Positive	Negative	Negative	Positive
41	Positive	Negative	Positive	Negative	Negative	Positive
42	Positive	Negative	Positive	Negative	Negative	Positive
43	Negative	Negative	Positive	Negative	Negative	Positive
44	Positive	Negative	Positive	Negative	Negative	Positive
45	Negative	Positive	Positive	Negative	Negative	Positive
46	Positive	Negative	Negative	Negative	Negative	Positive

47	Positive	Positive	Positive	Negative	Negative	Positive
48	Positive	Positive	Negative	Negative	Negative	Positive
49	Positive	Negative	Negative	Negative	Negative	Positive
50	Positive	Negative	Negative	Negative	Negative	Positive
51	Negative	Negative	Negative	Negative	Negative	Positive
52	Positive	Negative	Negative	Negative	Negative	Positive
53	Positive	Negative	Negative	Negative	Negative	Positive
54	Positive	Negative	Negative	Negative	Negative	Positive
55	Positive	Negative	Negative	Negative	Negative	Positive
56	Negative	Negative	Negative	Negative	Negative	Positive
57	Negative	Positive	Negative	Negative	Negative	Positive
58	Positive	Positive	Negative	Negative	Negative	Positive
59	Negative	Negative	Negative	Negative	Negative	Positive
60	Positive	Positive	Negative	Negative	Negative	Positive
61	Positive	Negative	Positive	Negative	Negative	Positive
62	Negative	Negative	Positive	Negative	Negative	Positive
63	Negative	Negative	Positive	Negative	Negative	Positive
64	Negative	Positive	Positive	Negative	Negative	Positive
65	Negative	Negative	Positive	Negative	Negative	Positive
66	Negative	Negative	Positive	Negative	Negative	Positive
67	Negative	Negative	Negative	Negative	Negative	Positive
68	Positive	Negative	Negative	Negative	Negative	Positive
69	Positive	Negative	Negative	Negative	Negative	Positive
70	Positive	Negative	Positive	Negative	Negative	Positive
71	Positive	Negative	Negative	Negative	Negative	Positive
72	Negative	Negative	Negative	Negative	Negative	Positive
73	Positive	Negative	Negative	Negative	Negative	Positive
74	Negative	Negative	Positive	Negative	Negative	Positive
75	Positive	Positive	Negative	Negative	Negative	Positive
76	Positive	Negative	Negative	Negative	Negative	Positive
77	Positive	Negative	Negative	Negative	Negative	Positive
78	Positive	Positive	Negative	Negative	Negative	Positive
79	Positive	Negative	Negative	Negative	Negative	Positive
80	Positive	Negative	Negative	Negative	Negative	Positive
81	Positive	Negative	Negative	Negative	Negative	Positive
82	Positive	Negative	Negative	Negative	Negative	Positive
83	Positive	Negative	Negative	Negative	Negative	Positive
84	Positive	Negative	Negative	Negative	Positive	Positive
85	Negative	Negative	Negative	Negative	Negative	Positive
86	Negative	Positive	Negative	Negative	Negative	Positive
87	Positive	Negative	Negative	Negative	Negative	Positive
88	Negative	Positive	Negative	Negative	Negative	Positive
89	Negative	Negative	Negative	Negative	Negative	Positive
90	Positive	Negative	Negative	Negative	Negative	Positive
91	Positive	Negative	Negative	Negative	Negative	Positive
92	Positive	Negative	Negative	Negative	Negative	Positive
93	Negative	Positive	Negative	Negative	Negative	Positive
94	Positive	Positive	Negative	Negative	Negative	Positive
95	Negative	Positive	Negative	Negative	Negative	Positive
96	Negative	Positive	Positive	Negative	Negative	Positive

97	Positive	Negative	Positive	Negative	Negative	Positive
98	Negative	Positive	Positive	Negative	Negative	Positive
99	Positive	Positive	Positive	Negative	Negative	Positive
100	Positive	Positive	Positive	Negative	Negative	Positive

References

#1 Khordadmehr M, Sazmand A, Almasi P, Shahbazi P, Ranjbar V, Otranto D, Hemphill A. Natural infection with *Toxoplasma gondii, Neospora caninum* and *Sarcocystis* species in domestic pigeons (*Columba livia domestica*) in Iran. Comp Immunol Microbiol Infect Dis. 2023 Feb;93:101946. doi: 10.1016/j.cimid.2023.101946. Epub 2023 Jan 5. PMID: 36621271.

#2 Khordadmehr M, Sazmand A, Oryan A, Almasi P, Ranjabr R, Hemphill A, Otranto D. Respiratory and intestinal zoonotic cryptosporidiosis in domestic pigeons (*Columba livia domestica*) in Iran. Iran J Vet Res. 2024
